# Interview with Dr. Errikos Maslias - 8^th^ European Congress on Neurorehabilitation in conjunction with the 20^th^ Congress of the Society for the Study of Neuroprotection and Neuroplasticity

**DOI:** 10.25122/jml-2026-1001

**Published:** 2026-01

**Authors:** Stefana-Andrada Dobran, Alexandra Gherman

**Affiliations:** 1 RoNeuro Institute for Neurological Research and Diagnostic, Cluj-Napoca, Romania; 2 Sociology Department, Babes-Bolyai University, Cluj-Napoca, Romania

**Interviewee: Dr. Errikos Maslias**

**Interviewer: Ms. Stefana-Andrada Dobran**

Dr. Errikos Maslias is a neurologist and clinical researcher at Lausanne University Hospital (CHUV), serving as Chef de Clinique in the University Service of Neurorehabilitation. He specializes in the intensive rehabilitation of brain injuries and disorders of consciousness. Since 2023, he has also been a clinical study investigator with the NeuroDigital@NeuroTech group and the Laboratory of Acute Neurorehabilitation (LNRA). His research centers on applying digital health technologies to enhance seizure detection, diagnosis, and early neuroprognostication in disorders of consciousness, as well as advancing precise neurorehabilitation and neuroprognostication for brain injury patients through novel brain connectivity approaches and large language models (LLMs).

**S.D.: Hello, Dr. Erikos Maslias and welcome to the 8^th^ European Congress of Neurorehabilitation (ECNR) in conjunction with the 20^th^ Congress of the Society for the Study of Neuroprotection and Neuroplasticity. The ECNR brings together scientific and clinical communities. What do you believe is the unique role it plays in bridging the gap between research and daily patient care in neurorehabilitation?**

**
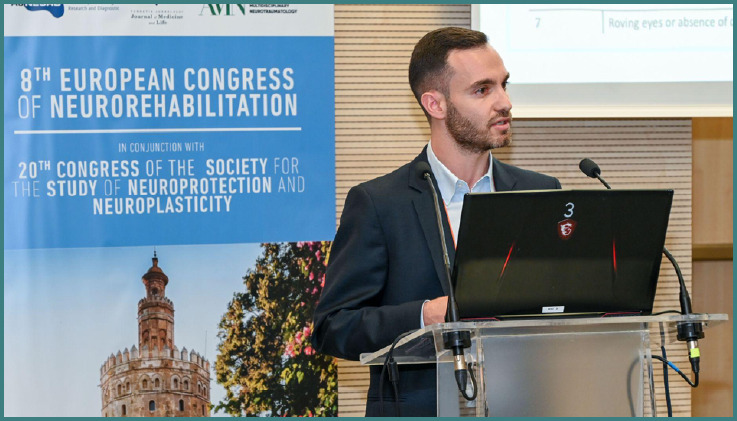
**

E.M.: Thank you, I'm very glad to be here. I believe that the clinical and research sides of medicine are deeply interconnected. Research often originates from questions that emerge in everyday clinical practice, and then, in return, clinical practice is improved by research findings. I believe that inter-connection is crucial. The EFNR Congress plays a key role in fostering this connection by bringing together professionals from diverse domains and specialties—some primarily focused on research, others on clinical practice, and some who combine both. This is an essential way to bridge the gap between the clinic and the research laboratory.

**S.D.: Considering your specialty, what future developments do you envision for the complex multidisciplinary field of neurorehabilitation?**

E.M.: It is a very exciting time for neurorehabilitation. We are seeing major improvements and new technologies, such as non-invasive brain stimulation, robotics, wearables, virtual reality, artificial intelligence, but we also see important paradigm shifts—for instance, the push to start neurorehabilitation earlier and more intensively. It’s a very exciting period.

For future directions, we should indeed start rehabilitation earlier and more intensively, though this can be sometimes challenging because it requires a multidisciplinary approach. The implications differ for physiotherapy, occupational therapy, speech and language therapy, neuropsychology, doctors and for nursing. So, everyone needs to adapt its practices to this paradigm shift.

**S.D.: Is there a specific technology that you are excited to see in the future?**

E.M.: Of course: artificial intelligence. I think it has a lot of potential. It can handle enormous amounts of data and identify patterns that would be impossible for human to detect on their own. For example, in disorders of consciousness, artificial intelligence could detect more subtle clinical signs during clinical examination—signs that are often missed by the human eye. That, I think, could really improve our practice in the intensive care unit as well.


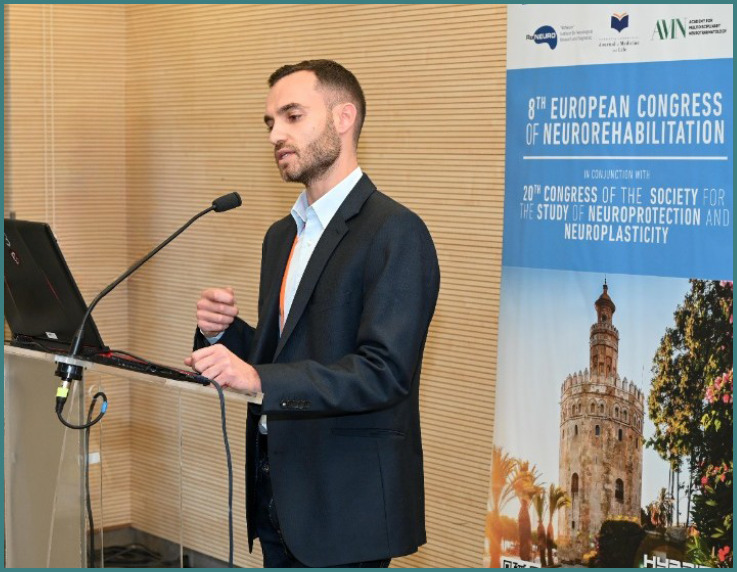



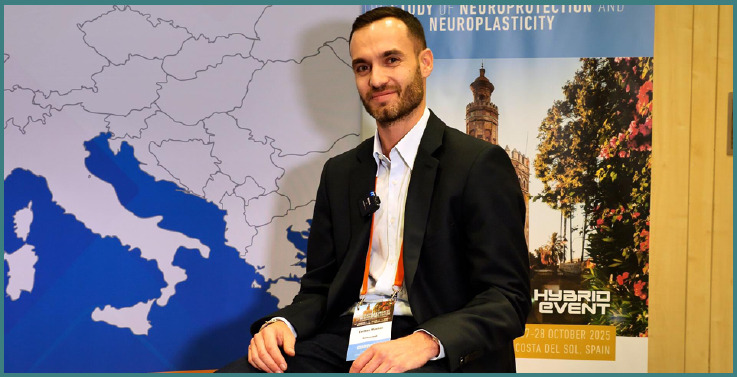


**S.D.: What is, from your perspective, the most challenging future development in neurorehabilitation and how can the EFNR come closer to this endeavor to bridge the gap?**

E.M.: I think that the challenge is quite closely related to the progress we’ve made. We now have a lot of technology; we rely heavily on neuroimaging and paraclinical techniques. The challenge is to remain clinical—to really focus on the clinical assessment of our patients. This is something that should also be taught to young doctors starting in the specialty, like myself. What the EFNR could do is offer workshops on clinical practice and clinical knowledge, focusing on how to properly examine patients with brain lesions—and specifically, patients with disorders of consciousness as well.

**S.D.: Your career path combines clinical neurology in neurorehabilitation and digital technologies. Based on this perspective, what do you see as the most critical element that should be solved or addressed?**

E.M.: I think that in these different fields, we face different challenges. To address these challenges, international collaboration is needed. We should collaborate more across countries to establish common definitions, common protocols for patient examination, and to conduct more collective studies—multicentric studies with larger patient populations. Neurorehabilitation studies are often limited for various reasons, so this is where we should concentrate our efforts to build the future of neurorehabilitation for both research quality and patient care.

